# The Small Molecule NLRP3 Inflammasome Inhibitor MCC950 Does Not Alter Wound Healing in Obese Mice

**DOI:** 10.3390/ijms19113289

**Published:** 2018-10-23

**Authors:** James S. Lee, Avril A. B. Robertson, Matthew A. Cooper, Kiarash Khosrotehrani

**Affiliations:** 1 UQ Diamantina Institute, Translational Research Institute, The University of Queensland, Wooloongabba, QLD 4102, Australia; j.lee@uq.edu.au; 2 School of Chemistry and Molecular Biosciences, The University of Queensland, St. Lucia, QLD 4072, Australia; a.robertson3@uq.edu.au; 3 Institute for Molecular Bioscience, The University of Queensland, St. Lucia, QLD 4072, Australia; m.cooper@uq.edu.au

**Keywords:** NLRP3, MCC950, chronic wound healing, small molecule inhibition

## Abstract

The incidence of chronic wounds is escalating, and the associated healing process is especially problematic in an aging population with increased morbidity. Targeting increased inflammation in chronic wounds is a promising but challenging therapeutic strategy. Indeed, inflammation and especially macrophages are required for wound healing. As the NLRP3 inflammasome has been implicated with various other inflammatory diseases, in this study, we used MCC950—a selective NLRP3 small molecule inhibitor—on murine models of both acute and chronic wounds. This molecule, while tested for other inflammatory conditions, has never been investigated to reduce topical inflammation driving chronic wounds. We found that there were no significant differences when the treatment was applied either topically or orally in wild-type C57Bl/6 mice and that it even impaired wound healing in obese mice. The treatment was also unable to improve re-epithelialisation or angiogenesis, which are both required for the closure of wounds. We are inclined to believe that MCC950 may inhibit the closure of chronic wounds and that it does not alter wound-associated macrophage polarisation.

## 1. Introduction

In the expanding population, chronic wounds are a significant issue and are associated with comorbidities such as an obesity, diabetes and aging. Varying interventions have been trialled in order to induce wound closure, such as negative pressure, hyperbaric oxygen, growth factor supplementation and acellular scaffold therapies, all with varying success due to the large heterogeneity in chronic wounds [1]. Despite these varying methods, data from the Centres for Disease Control and Prevention (CDC) identified that there were still five lower-extremity amputations per 1000 people with diabetes in 2014 [2]. With a 5-year post-operative mortality rate of 50%, new methods of treating chronic wounds need to be further explored.

Current research indicates that a majority of these ulcers develop due to impaired vascularisation, with increased recruitment of inflammatory leukocytes [3]. Under normal healing conditions, early recruited monocytes differentiate into inflammatory macrophages, which then shift their phenotype into a pro-healing, anti-inflammatory macrophage [4]. However, it is believed that prolonged accumulation of inflammatory macrophages due to haemostasis and iron overloading initiates an inflammatory phenotype in the lower limbs of chronic venous leg ulcer patients [5]. Multiple other studies have identified that interventions which are able to change the polarisation state of macrophages from inflammatory to noninflammatory/pro-healing are able to significantly improve wound closure [6]. However, if the intervention results in a depletion of macrophages, this will result in deleterious outcomes [7].

Previous reports have highlighted the importance of inflammasomes as mediators of inflammation in macrophages, especially in the context of chronic diseases [8]. The nod-like receptor family protein 3 (NLRP3) inflammasome is the most well characterised inflammasome, and its formation is triggered by a diverse number of pathogen-associated molecular patterns (PAMPS) as well as danger-associated molecular patterns (DAMPS), both of which would be present in wounds. When triggered, NLRP3 protein binds to the adaptor protein ASC and subsequently Caspase-1. Once formed, this NLRP3 inflammasome complex activates caspase-1, which initiates proteolytic cleavage of pro-IL-1β and pro-IL-18 into functional pyrogenic cytokines IL-1β and IL-18. A small molecule inhibitor termed MCC950 has been published as a potent and selective NLRP3 inflammasome inhibitor [9]. We used this molecule to test whether MCC950 was able to improve wound closure in an acute setting using C57Bl/6 mice and then in a chronic wound model using obese mice. Termed Ob/Ob, these mice replicate a phenotype similar to human patients, with delayed wound healing and chronic wounds [10]. Although they are obese, they do not develop a diabetic phenotype [11]. As we have initially found no significant results with the use of MCC950 in wound healing, this paper has been presented as a short communication.

## 2. Results

### 2.1. Topical Treatment of MCC950 Was Insufficient to Promote Wound Closure in WT and Obese Mice

**Figure 1:**
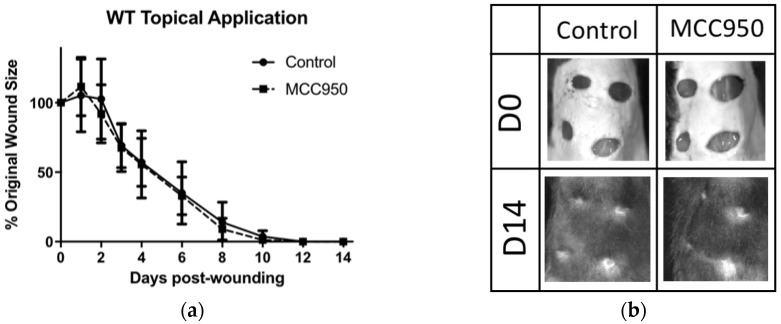

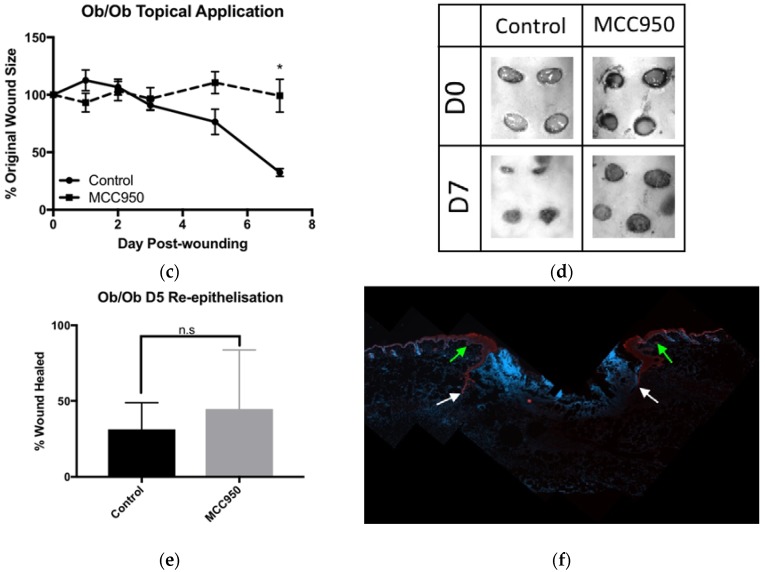
MCC950 appears to inhibit wound closure in diabetic mice when administered topically. (**a**) Comparing topically applied MCC950 vs. control, there was no significant difference in the rate of wound closure. (**b**) Representative photographs of wound closure on C57Bl/6 mice. (**c**) Comparing topical administration of MCC950 vs. control on obese mice with chronic wounds, the control mice had improved wound healing at D5 compared to MCC950. (**d**) Representative photographs of wound closure on Ob/Ob mice. (**e**) Histogram comparing re-epithelialisation between MCC950 and controls in Ob/Ob mice at D5 using keratin-14 staining. (**f**) Representative photomicrograph showing epithelial migration stained with keratin-14 (red) over the wound bed, stained with 4′,6-diamidino-2-phenylindole (DAPI) (blue). The last hair follicle before the opening of the wound (green arrows) was used to determine how far the epithelium had migrated along the wound bed (white arrows). N.s. = Not significant.

C57Bl/6J mice were treated topically with MCC950 [1 mg/dose] in 30% pluronic gel, daily from D0 to D4 post-wounding, based on previous studies using small molecule inhibitors topically [12,13]. No significant difference in wound surface was found between MCC950 and gel alone (control) groups (Figure 1a,b). As C57Bl/6J mice are known as a naturally fast-healing strain, we thought we might find more of a difference in models of delayed wound healing. We therefore used Ob/Ob mice older than six months as these display a chronic wound healing phenotype. Unexpectedly, topical MCC950 significantly impaired wound closure compared to control (Figure 1c,d) when measured macroscopically. However, upon further examination of the wounds using keratin-14 immunostaining to measure wound re-epithelialisation at D5, no significant difference was found between the groups (Figure 1e,f).

### 2.2. MCC950 Does Not Alter Wound-Associated Macrophage Polarisation

**Figure 2:**
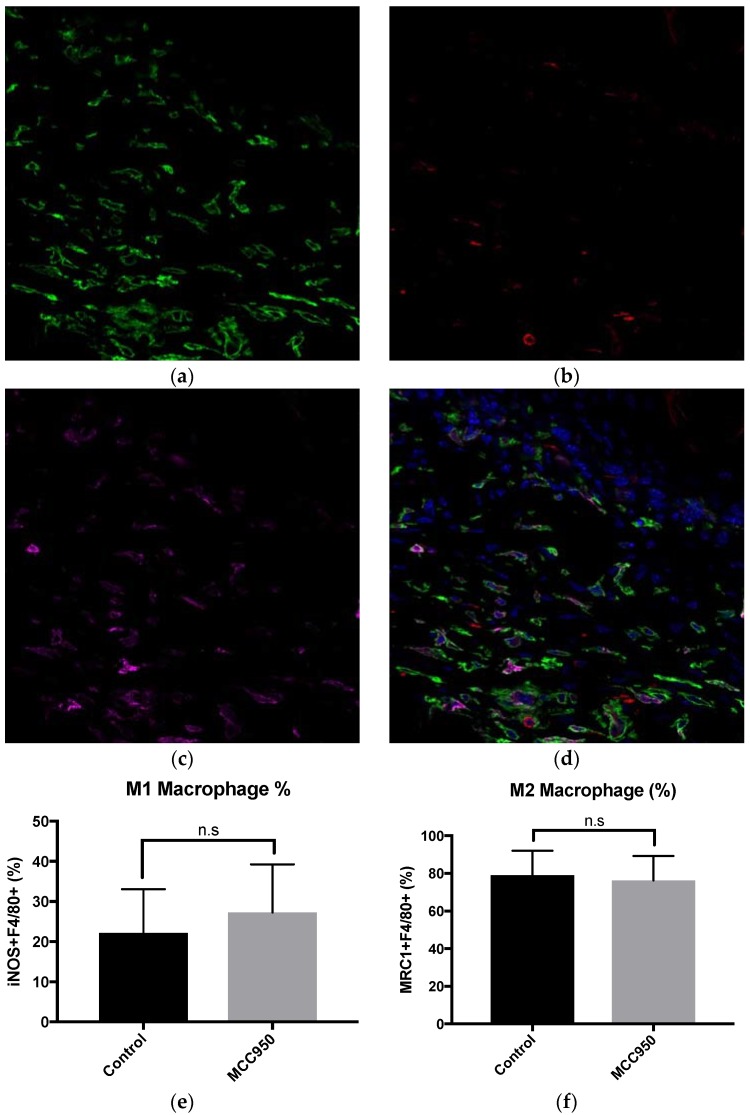
MCC950 does not alter wound associated macrophage populations. (**a**) Representative photomicrograph of macrophages stained with F4/80 (green). (**b**) iNOS to stain for M1 macrophages (red). (**c**) MRC1 to stain for M2 macrophages (purple). (**d**) A merged image of all antibodies plus DAPI for nuclear staining (blue). (**e**) No significant difference in M1 macrophage percentage between control and treated groups. (**f**) No significant difference in M2 macrophage percentage between control and treated groups. Error bars = SD. N.s. = Not significant. Scale bar = 100 μm.

As IL-1β is a pro-inflammatory cytokine associated with the inflammasome, we decided to next investigate the effect of MCC950 administration on macrophage polarisation in wounds. Using the Ob/Ob mouse wounds at D5 from the previous experiments, we used immunofluorescent staining against macrophages (F4/80) with co-staining against proinflammatory M1 (iNOS+) or pro-wound healing-associated M2 (MRC1+) macrophages between groups at D5 (Figure 2a–f). We found no significant differences in macrophage numbers or in polarisation for either M1 or M2 between treated groups. 

### 2.3. Oral Treatment of MCC950 Was Insufficient to Promote Wound Closure in Obese Mice

**Figure 3:**
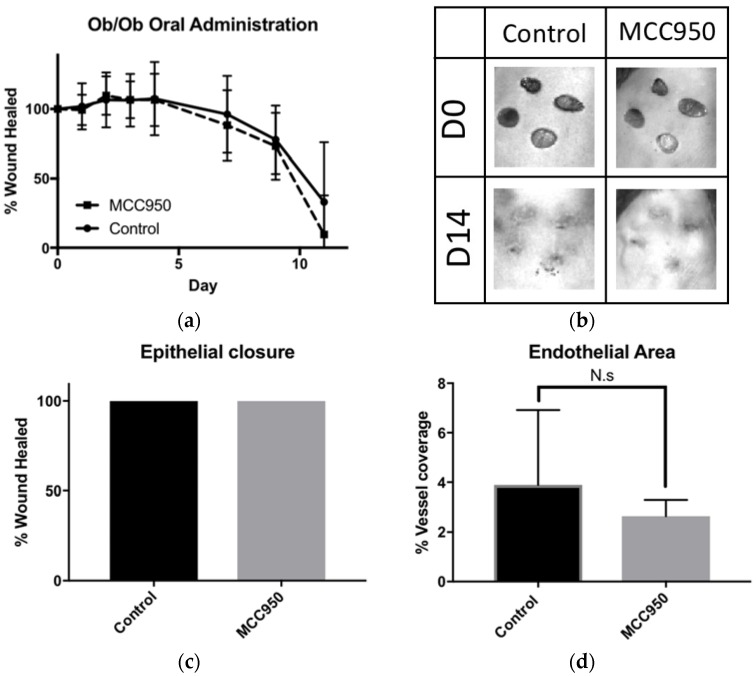
Oral administration of MCC950 does not significantly improve chronic wound healing. (**a**) Comparing orally administered MCC950 to control, there was no significant difference in the rate of macroscopic wound healing. (**b**) Representative photographs of wound closure on Ob/Ob mice. (**c**) Histogram comparing wound re-epithelialisation at D14 between groups using keratin-14 immunofluorescent staining. (**d**) Histogram comparing endothelial area between groups at D14 using CD31 immunofluorescent staining.

We next decided to investigate a systemic, rather than local, treatment of MCC950 as this method has been previously described to induce sufficient NLRP3 inhibition in cases of experimental autoimmune encephalomyelitis [9]. We therefore added MCC950 into the drinking water of obese mice at a concentration of 0.3 mg/mL to take ad libitum, repeating our previous data showing efficacy of MCC950 to inhibit NLRP3 activation and subsequent IL-1β production at 20 mg/kg [9]. We measured wound closure both macroscopically and histologically. Macroscopically, we found no significant difference between treated and untreated groups (Figure 3a,b). We also did not find any significant differences histologically as all wounds were completely covered with an epidermis by D14 measured with keratin-14 staining (Figure 3c). As angiogenesis is a major step in wound healing, we next looked at CD31+ vessel coverage in wound granulation tissue. There were no significant differences between MCC950-treated mice vs. control in angiogenesis at D14 (Figure 3d). 

## 3. Discussion

Wound healing is a delicate balance of inflammation and growth factors, each of which is essential at specific phases of the healing process. Indeed, many previous studies have identified the ablation of specific leukocyte populations, such as neutrophils, as beneficial for wound healing [14], whereas the ablation of macrophages from wounds results in impaired closure [15,16]. While macrophages have been described as “essential” for effective wound healing, their presence is mostly required for their pro-wound healing and remodelling properties rather than their early inflammatory functions [17]. Therefore, current research has focused on inhibiting the inflammatory phase without compromising the pro-healing properties of these cells.

As the inflammasome is a key player in the induction of inflammation and is associated with many inflammatory disorders [8], we expected MCC950 to improve wound closure in the context of chronic wounds by preventing excess inflammation. However, we failed to observe any significant change in wound healing at all, with possible impairment of wound healing in one of our treatment groups. We also failed to observe any significant effects through histological experiments, indicating that the macroscopic observation may have just been an artefact due to slightly improved wound contraction. Macrophages are a key player in the regulation of wound healing, where having an increased inflammatory profile from diabetes in theory should impair wound healing. This impaired wound healing is observed when comparing WT mice to Ob/Ob mice, where WT mice heal within seven days and Ob/Ob mice take up to two weeks to heal wounds of the same size [18]. MCC950 has been previously described to have no effect on obesity or diabetes in a model of *db/db* diabetic mice [19]. Therefore, to study the effects of inflammasome-driven inflammation in chronic wound healing, the *db/db* model seemed ideal to test the effects of MCC950 on diabetic wounds, without affecting the diabetic phenotype itself. In these experiments, we used Ob/Ob mice that have a deficiency in the leptin protein rather than a deficiency in the leptin receptor of the db/db mice used in the previous study. These mice have different genetic backgrounds, and while the db/db mice develop a strong obese and diabetic phenotype, the Ob/Ob mice are more resistant to the diabetic phenotype with only mild hyperglycemia [11]. 

Recently, in accordance with our findings, NLRP3 knockout mice have been described to have impaired wound healing [20,21], albeit not in a chronic wound healing model. Although, Lee et al. [22] identified that monocyte-derived macrophages from diabetic patients expressed higher levels of NLRP3 and its associated inflammatory cytokines, it is possible that inhibition of this pathway may seem too deleterious for the process of healing. Alternatively, the reduction of NLRP3-induced inflammation is not significant compared to other proinflammatory cytokines, such as TNFα, IL-6 or iNOS, present in chronic wounds [23]. Chronic wounds are a complex, multifactorial disease with many different facets regarding inflammation, presence of growth factors, venous insufficiency and vascularisation. This may be why we did not observe any significant differences in mice treated with MCC950 because NLRP3 inhibition alone was insufficient to reduce overall inflammation in the chronic wounds.

NLRP3 has also been described as important for angiogenesis, in tumour models [24] and in matrigel plugs [25]. As angiogenesis is a critical part of wound healing, especially in chronic wounds [26], we aimed to identify if we could recapitulate the results found in previously published papers. What we observed was a trend toward reduced angiogenesis in our mice at D14 wounds; however, this difference was not statistically significant. Further studies investigating long-term effects on endothelial-to-mesenchymal transition (EndMT) could also be useful in studying long-term scar formation due to the reduction in NLRP3-associated inflammation [27].

The rising cost of chronic wound treatments and therapies is a massive economic strain on healthcare systems. The development of easy-to-manufacture small molecules as an alternative to expensive antibody therapies is a new avenue that aims to lessen the burden of rising healthcare costs. While the novel, cost-effective small molecule inhibitor MCC950 has shown efficacy in the treatment of other inflammatory conditions, unfortunately in this case, it appears that inhibiting the NLRP3 inflammasome using the small molecule MCC950 does not significantly affect wound closure. This may simply be due to the fact that the NLRP3 inflammasome itself does not play a significant role in macrophage polarisation in a chronic wound healing scenario.

## 4. Materials and Methods

### 4.1. Mice

All mice were bred on a C57B/6J background. C57Bl/6J and Ob/Ob mice were wounded after 6 months of aging to promote the chronic wound healing phenotype. Equal numbers of males and females were used. All experiments had 5 control vs. 5 treated mice per group per timepoint. Four wounds per mouse were analysed and averaged per mouse for statistical analysis. All mice were treated in accordance with institutional ethics approvals and guidelines for the care of experimental animals, performed under The University of Queensland, Research and Innovation, Animal Welfare Unit, ethics number UQCCR/460/15/STARTUP, Start date 28 November 2015 to 28 November 2018.

### 4.2. Experimental Procedures

Mice were wounded as per previous literature [28]. Mice were shaved and had Veet depilatory cream applied to remove excess hair. Four 6mm, full-thickness wounds were created using a 6mm punch biopsy and wounds were left to heal without bandaging or covering. MCC950 was first applied topically at a dose of 1 mg/day in 30% F-127 Pluronic gel dissolved in phosphate-buffered saline (PBS). The second experiment used MCC950, taken orally at a dose of 0.3 mg/mL in drinking water ad libitum, giving a dose of approximately 20 mg/kg/day in replication of previous experiments performed and published [19]. MCC950 was supplied by the Institute for Molecular Bioscience, University of Queensland, Australia.

Wounds were photographed every second day using a standardized xenogen live imaging charge-coupled device camera (CCD) system. Wound images were analysed using ImageJ software, where the edge of the wound was traced and measured as an area and was calculated as a percentage of the original wound area based on D0 wounds.

### 4.3. Histology—Frozen Sections

Wounds were harvested at D5, D7 and D14 post-wounding. Wounds were fixed in 4% paraformaldehyde for 2 h and cryoprotected in gradients of sucrose up to 30% in PBS until the tissue sank. Wounds were sectioned through their centre and embedded cut side down in Sakura Tissue-Tek™ OCT on an isopentane/dry ice mix. Frozen section slides were cut at 8 µm on Superfrost Plus slides and stored at −30 °C until use.

### 4.4. Histology—Paraffin Section

Wounds were harvested at D14 post-wounding. Wounds were fixed in 4% paraformaldehyde for 24 h at 4 °C. Wounds were sectioned through the centre, then dehydrated and paraffin-embedded in a Leica HistoCore Pearl tissue processor (Leica Biosystems, Wetzlar, Germany) for 12 h. Sections were cut at 4 µm on Superfrost Plus slides (Thermofisher, Waltham, MA, USA).

### 4.5. Immunofluorescence

Frozen section slides were rehydrated in PBS-Tween 20 0.1% (PBS-T) for 5 min. Slides were blocked in 10% normal goat serum, 1% bovine serum albumin (BSA) (Sigma Aldrich, St. Louis, MO, USA) and PBS-T for 15 min at room temperature. Primary antibodies—keratin-14 (EP1612Y, Abcam, Cambridge, UK) 1:1000, CD31 (MEC13.3, BD Bioscience, Franklin Lakes, NJ, USA) 1:200, F4/80 (CI-A3-1, Bio-Rad, Hercules, CA, USA) 1:200, iNOS (Polyclonal, Abcam, Cambridge, UK) 1:100 and LYVE-1 (Polyclondal, Abcam, Cambridge, UK) 1:200—were diluted in blocking solution and incubated overnight at 4 °C. Secondary antibodies were a combination of Alexafluor-A488, A568 or A647 (Thermofisher, Waltham, MA, USA) 1:500 dilution in 3% BSA and PBS-T. Slides were counterstained with DAPI (Thermofisher, Waltham, MA, USA) and imaged on a Carl Zeiss LSM510 Meta (Carl Zeiss, Jena, Germany). 

## Abbreviations

D”x”	Day “X” post-wounding
DAMPS	Danger-associated molecular patterns
PAMPS	Pathogen-associated molecular patterns
PBS	Phosphate-buffered saline
NALP3	NACHT, LRR and PYD domains-containing protein 3
WT	Wild-type
Ob/Ob	Obese mouse
